# Predictive Value of Fractional Exhaled Nitric Oxide (FeNO) in the Diagnosis of Asthma for Epidemiological Purposes—An 8-Year Follow-Up Study

**DOI:** 10.3390/arm92010006

**Published:** 2024-01-04

**Authors:** Kamil Barański

**Affiliations:** Department of Epidemiology, School of Medicine in Katowice, Medical University of Silesia in Katowice, 40-055 Katowice, Poland; kbaranski@sum.edu.pl

**Keywords:** asthma, FeNO, prediction, follow-up study, epidemiology

## Abstract

**Highlights:**

**What are the main findings?**

- It seems that children with asthma-like symptoms and with elevated FeNO values have a higher probability of a diagnosis of asthma in the future.
- Early-age school children without asthma-like symptoms and with a FeNO of <35 ppb are unlikely to present in the next 8 years asthma-like symptoms.

**What is the implication of the main finding?**

- Children who underwent the FeNO measurement in community settings should be investigated in clinical conditions for further verification.

**Abstract:**

At the population level, respiratory symptoms in children can be estimated cross-sectionally. However, such methods require additional objective support parameters, such as the measurement of fractional exhaled nitric oxide (FeNO). The aim of the present study was to analyze if the FeNO value measured at baseline can have a predictive value for asthma-like symptoms after 8 years of measurement. Methods: The follow-up included 128 (out of 447) children, 70 girls and 58 boys. The FeNO was measured at baseline only. The prevalence of asthma-like symptoms was measured with the adopted version of the ISAAC questionnaire. Results: After 8 years of FeNO measurement, 5 new cases of asthma, 2 cases of attacks of dyspnoea, 1 case of wheezy in the chest, and 18 cases of allergic rhinitis occurred. The FeNO values, measured at the baseline of the study, for new cases of the above diseases were 53.4 ± 75.9 ppb, 11 ± 1.5 ppb, 12.0 ppb, and 16.3 ± 12.4 ppb, respectively. The best diagnostic accuracy parameters were found in the new cases of asthma, where the sensitivity was 40.0%, the specificity was 98.6%, and the AUC was 66.6%. The diagnostic odds ratio was 46.9 when considering the FeNO cut-off >35 ppb. Conclusions: The FeNO measurement is a fair method for asthma prognosis in early school-aged children with asthma-like symptoms measured on the population level but requires further confirmation at the clinical level with more accurate diagnostic tools.

## 1. Introduction

In Poland, the prevalence of asthma in children is around 9% [1,2]. Asthma-like symptoms, such as wheeziness in the chest, dyspneic attacks, atopic rhinitis, and atopic dermatitis, occur more frequently in school-aged children. Axiomatically, this suggests that some of the symptoms are predictive of more serious health issues or signify an underdiagnosis of respiratory diseases. In Poland, the prevalence of allergic rhinitis increased from 51.2% to 62.1% between 2008 and 2015 and decreased to 52% in 2018 in children aged 7–8 years. In young adults (aged 16–17), the prevalence of allergic rhinitis decreased from 63.9% to 35.8% in the same period of 2008–2018 [2]. Regarding the prevalence of asthma in children aged 7–10 years, asthma was noted in 3.4% to 12.6% of children between 1993 and 2014 [3]. The prevalence of attack of dyspnoea in the last 12 months was reported in 11.2% of children aged 6–15 and the episode of wheezing was 9.6% in the last 12 months in 2018 [4].

Currently, most studies use questionnaires for assessing asthma-like symptoms at the population level, which is a commonly accepted method [5]. However, the accuracy of questionnaires might be unsatisfactory. The International Study of Asthma and Allergies in Childhood Questionnaires (ISAAC) questionnaire, used for respiratory symptoms assessment in children, has an accuracy from 26% to 76.9% depending on the assessed symptoms [6]. Respiratory diagnosis at the populational level needs supportive and more objective methods of assessment. One such method that is safe, inexpensive (depending on the country and health system), and easy to perform is a measurement of fractional exhaled nitric oxide (FeNO), a marker of eosinophilic airway inflammation [7]. Additionally, this biomarker is commonly used for asthma diagnosis and managing the treatment process of asthma in children [8]. Current literature shows that FeNO is linearly associated with the age in children aged 6–14 years; however, it is not fully understood how FeNO varies within the physiological changes across age and sex [9]. Moreover, the predictive value of FeNO has not yet been fully elucidated in the literature [10].

The aim of the study was to assess the predictive value of the FeNO measurement in school-aged children in relation to respiratory symptoms after 8 years from the first FeNO measurement.

## 2. Materials and Methods

This study was initiated in 2014. The baseline of the study included 506 children from the randomly selected primary classes in four schools (in cities: Bytom, Chorzów, Tychy, and Zabrze) in the Silesia Voivodship, Poland. The acceptable FeNO measurement was made in 447 children (an 89% success rate). Moreover, only children in whom parents or legal guardians responded to the modified version of the Study of Asthma and Allergies in Childhood (ISAAC) questionnaire were included in the study [11].

The core variables, such as asthma, attack of dyspnea, symptoms of wheezy, allergic rhinitis, and atopic dermatitis, were assessed to the following questions:

1. Variable “asthma” was assessed on the response to the question: Has your child ever had asthma diagnosed by a physician?
2. Variable “wheezy” was assessed in response to the question: Has your child had wheezing or whistling in the chest in the past 12 months?
3. Variable “attack of dyspnea” was assessed on the response to the question: Has your child ever had an attack of dyspnea in the past 12 months?
4. Variable “allergic rhinitis” was assessed on the response to the question: Has your child ever had hayfever in the past 12 months?
5. Variable atopic dermatitis was assessed on the response to the question: Has your child ever had atopic dermatitis in the past 12 months?

The FeNO measurement (NIOX MINO device, Circassia, Stockholm, Sweden) was performed according to ERS/ATS recommendations [12]. The tests were performed by a trained and certified researcher. All children and parents were informed to avoid drinking and eating 1 h prior to FeNO measurement. The results of the FeNO measurement were expressed in part per billion (ppb). The measurement of FeNO was conducted on the second day of the week to decrease the risk of the impact of exposure to tobacco smoke (positive smoking status of parents).

The follow-up was conducted in 2022 and included 128 (out of 447) children due to limitations regarding parents’ or legal guardians’ informal consent and response, which was via traditional post with the questionnaire. The FeNO measurement was performed only at baseline in 2014. The reason for assessing children after 8 years from the baseline is that children aged 6–7 and 13–14 are at the highest risk (the prevalence of asthma is relatively high in these group ages) of developing asthma. The mentioned group ages covered the group ages of the ISAAC study.

The structure of this study had the following order:

1. Invitation to the study and assessment of respiratory status according to the questionnaire;
2. FeNO measurement;
3. Questionnaire (traditional letter—self-addressed stamped envelope) after 8 years from their FeNO measurement.

The letter included information about the invitation for a FeNO measurement.

## 3. Statistical Analysis

The results for quantitative variables were expressed as the arithmetic mean and standard deviation. For the qualitative variables, the number (frequency) of cases was used. The differences in FeNO between the four defined groups (group 1: baseline negative and follow-up negative, group 2: baseline negative and follow-up positive, group 3: baseline positive and follow-up negative, and group 4: baseline positive and follow-up positive) were calculated with the Kruskal–Wallis test with correction for multiple comparisons. The normal distribution of FeNO in total and the subgroups was assessed with the Shapiro–Wilk test. The Wilcoxon test was used in the case of assessing differences in FeNO according to sex.

The diagnostic accuracy was measured with the diagnostic odds ratio (DOR), which does not depend on disease prevalence.

The DOR was calculated with the following formula [13]:

DOR = Sensitivity(1 − Sensitivity) + (1 − Specificity)Specificity(1)

The frequency of true positives (TP) and false negatives (FN) was described according to asthma-like symptoms while considering the three defined thresholds of FeNO values suggested by ATS/ERS and other researchers (20, 25, 35 ppb) as well. [8,14]. Moreover, the diagnostic accuracy formulas included sensitivity (the ability of the test to detect people with the disease) and specificity (the ability of the test to detect people without the disease [15]. The area under the curve (AUC) was calculated to present FeNO accuracy as a continuous variable [16] for each diagnosis/symptom. The positive predictive value was the number of true positives divided by the sum of the number of true positives and false positives, while the negative predictive value was the number of true negatives divided by the sum of true positives and false positives.

All analyses were performed only in children who participated in the baseline and follow-up studies (n = 128) using the SAS statistical package (SAS Institute Inc., Cary, NC, USA, version 9.4).

## 4. Results

Reported respiratory symptoms (baseline vs. follow-up).

In the study, there were 128 children who participated in the follow-up. There were 45% (n = 58) boys and 55% (n = 70) girls. The children did not differ significantly according to age (*p* = 0.6) between sexes in the follow-up. 

Regarding the symptoms from the respiratory system, there were 92.9% (n = 119) children without any asthma at baseline and after follow-up, 5/128 children without asthma at baseline but reported asthma during the follow-up, 1 child had asthma at the baseline but denied asthma diagnosis at follow-up, and 3/128 children had asthma confirmed by a physician at the baseline of the study and at the follow-up of the study. 

Lack of asthmatic tendency (defined as symptoms of wheeziness in the chest and dyspnoea reported in the last 12 months) at baseline and at the follow-up was reported by 95% (n = 122) of parents; 1 child had no symptoms of asthmatic tendency at baseline and reported asthmatic tendency during follow-up, and 4/128 children had symptoms of asthmatic tendency at the baseline of the study and denied those symptoms during follow-up. One child had an asthmatic tendency at the baseline and at the follow-up.

The symptoms of dyspnoea during the last 12 months were not reported at the baseline or during follow-up in 96.8% (n = 124) of children, while no symptoms of dyspnea at the baseline but during follow-up were reported in 3/128 children, while in only 1/128 children, there were symptoms of dyspnea at the baseline but not during the follow-up. None of the children had symptoms of dyspnea during the day at baseline and at the follow-up of the study. 

The lack of occurrence of wheezing currently or in the past 12 months was reported in 86.7% (n = 111) of children, while no symptoms of wheezing at the baseline but during the follow-up were reported in 1/128 children. At baseline, but not during the follow-up, there were 12/128 children with wheezing episodes; in 4/128 children, the wheezing was reported at the baseline and during the follow-up of the study. The frequency of the symptoms at the baseline and during the follow-up, with the mean FeNO values measured only at baseline, are described in Table 1. The first column, column 1, describes children without symptoms (mentioned in the row) at the baseline and follow-up; the second column describes children without symptoms at the baseline but with symptoms at follow-up; the third column describes children with symptoms at the baseline but without symptoms at follow-up; and the fourth column describes children with symptoms at the baseline and follow-up.

Reported allergic symptoms (baseline vs. follow-up).

A lack of allergic rhinitis at the baseline and during the follow-up was noted in 63.2% (n = 81) of children; no symptoms of allergic rhinitis were reported at baseline but not during the follow-up in 14% (n = 18); the occurrence of allergic rhinitis at the baseline but lack of allergic rhinitis during follow-up was reported in 16.4% (n = 21); the occurrence of allergic rhinitis at the baseline and during follow-up was noted in 5/128 children. In 82% (n = 105) of children, there were no symptoms of atopic dermatitis at the baseline and during the follow-up, and no atopic dermatitis at the baseline but reported during follow-up was noted in 3/128 children. Atopic dermatitis was reported at the baseline but not during the follow-up and was noted in 10.1% (n = 13), while in 7/128 children, atopic dermatitis was reported at the baseline and the follow-up of the study; Table 1.

The accuracy of FeNO in children who developed asthma was calculated in contrast to children without any considered health burden (72 children). The sensitivity (SEN) was 40% (2/5), the specificity (SPE) was 81.9% (59/72), the positive predictive value (PPV) was 95.1% (59/62), and the negative predictive value (NPV) was 13.3% (2/15) for a FeNO cut-off of 20 ppb. The SEN for the recommended high FeNO cut-off (>35 ppb) was 40% (2/5), the SPE was 98.6% (71/72), the PPV was 66.7% (2/3), and the NPV was 95.9% (71/74). There were 18 new cases of allergic rhinitis: the SEN was 16.6% (3/18), the SPE was 81.9% (59/72), the PPV was 18.7% (3/16), and the NPV was 79.7% (59/74); for a FeNO cut-off of 20 ppb, the SEN was 11.1% (2/18), the SPE was 98.6% (71/72), the PPV was 66.6% (2/3), and the NPV was 81.6% (71/87); Table 2. Due to the low number of cases of attacks of dyspnea, wheezing, and atopic dermatitis, it was not valid to calculate the diagnostic accuracy. 

## 5. Discussion

The aim of this study was to examine the correlation between FeNO levels and the presence or absence of respiratory/allergic symptoms reported at the baseline and compare them with the reported presence or absence of respiratory/allergic symptoms at an 8-year follow-up in the same population.

The results of this study suggest that children aged 6–9 years with asthma-like symptoms and FeNO values of >35 ppb have the highest chance of having respiratory disease in the future. The diagnostic odds ratio resulted in almost 47 scores, which, according to the test value, has an extremely good predictive value [17]. In this study, other diagnostic indicators like AUC or sensitivity, specificity, and true-positive values do not support the value of the diagnostic odds ratio. The FeNO had the best accuracy in relation to asthma. The sensitivity was 40% with a 95% confidence interval (95% CI) ranging from 0 to 83% for each analyzed FeNO cut-off (>20 ppb, >25 ppb, and >35 pbb). The specificity was 81.9% (95% CI 73–90%), 94.4% (95% CI 89–100%), and 98.6% (95% CI 96–100%) for each FeNO cut-off. The true-positive indicators for FeNO cut-offs were 13.3% (95% CI 9–30%), 33.3% (95% CI 0–71%), and 66.6% (95% CI 27–100%), respectively. The results of the false-negative indicators for FeNO cut-offs were 95.1% (95% CI 90–100%), 95.7% (95% CI 91–100%), and 95.9% (95% CI 91–100%), respectively. Such findings are consistent with the results reported in systematic reviews and meta-analyses from other cross-sectional studies [18].

The other issue that cannot be omitted is the question if children with increased FeNO values were not yet diagnosed with asthma (underdiagnosis of asthma). From a total group of children (n = 447), there were 22 cases of asthma. In the group of children who participated in the follow-up part, at the baseline, there were four cases of asthma (mean FeNO: 33.0 ppb; range: 10–52 ppb) and eight cases of asthma (mean FeNO: 47.2 ppb; range: 6–186 ppb) during the follow-up. One child from baseline asthma had asthma excluded after 8 years, so in total, there were five new cases of asthma (mean FeNO: 53.4 ppb, range: 6–186 ppb). This subtly suggests that children with elevated FeNO levels (>34 ppb) have not been diagnosed by a physician. In the five new cases of asthma, two children had attacks of dyspnoea, three children had wheeziness in the chest, and in all five cases, there were symptoms of allergic rhinitis and atopic dermatitis. In the study performed by Caudri et., all the FeNO was used as a predictor of asthma in preschool children from the PIAMA cohort. It shows FeNO as a significant predictor of asthma-like symptoms such as wheezing and steroid use in the future [19]. However, the study was performed in the clinical field and in children with any asthma-like symptoms, so the conditions of the study differed from the methodology of the presented study. Moreover, the present study was performed in children who were free of respiratory symptoms as well. 

More studies focused on the accuracy of FeNO in the diagnosis of asthma rather than the prediction of FeNO in asthma diagnosis. Some studies showed that the FeNO (>15.8 ppb) diagnostic accuracy (area under the curve ROC = 0.53) was poor in children with symptoms suggesting asthma [20]. Contrasting results were shown in a study performed by Malberg. Children with probable asthma had higher FeNO concentrations in comparison to healthy controls. Furthermore, the same study showed discriminant accuracy of FeNO with a sensitivity of 86% and specificity of 92%; a solid result, especially since sensitivity and specificity are inversely related [21]. This corresponds with the conclusions suggested by Pijnenburg. Ostensibly, the measurement of FeNO in preschool children may be more accurate for asthma diagnosis in children and respiratory symptoms, particularly in atopic children [10]. Supportive findings for FeNO utility in school-aged children were found in the study performed by Kovesi. The authors concluded that FeNO levels reflect allergic conditions, including allergic asthma [22].

Contrastingly, in a prior study conducted by the author, the conclusion reached was that FeNO measurement was not an effective screening tool for pediatric asthma in a community setting [23]. However, the findings of the present study contradict this, indicating that children exhibiting asthma-like symptoms and elevated FeNO levels are at an increased risk of asthma, and these findings suggest the need for additional investigation. Additionally, these results align with the findings of a cross-sectional screening study conducted by Prasad et al. [14]. The predictive meaning of FeNO needs to be controlled according to the determinants of FeNO. In the study performed by See and Christiani, ethnicity, height, self-reported rhino-conjunctivitis, and household smoke exposure were responsible for 10.3% of the FeNO variability in children aged 6–11 [24]. According to their study, they based the symptoms from the respiratory system on the responses from the questionnaire. The lack of validated diagnosis probably decreased the explained variability of FeNO; however, their study reflected the study performed in the epidemiological conditions [23]. In the study performed by Garcia-Marcos, it was revealed that approximately 27% of the variability in FeNO levels could be explained. The 20% of the variability was explained by age, rhino-conjunctivitis, a positive skin prick test, and the removal of cats and/or dogs from the home environment [25]. The internal variability (within the group) of FeNO seems to be stable according to the current literature [26,27] and should not impact the differences between the status of children. 

It appears that the predictive significance of FeNO in the diagnosis of asthma is contingent upon several critical factors. Specifically, the FeNO level is influenced by numerous determinants that necessitate thorough elucidation [28,29]. Moreover, the exact reference FeNO values, like for spirometry, should be developed. The adjustment (reference FeNO values) is necessary to prove the applicability of FeNO in different age groups, such as children, teenagers, and probably adults. The current literature results imply that baseline FeNO levels seem to be a good predictor of a greater risk of moderate to severe asthma exacerbations, especially in uncontrolled asthma. These results enhance the significance of FeNO measurements in a community setting [30]. However, the published report titled: *Utility of Biomarkers in the Diagnosis and Monitoring of Asthmatic Children* by Xepapadaki et al. in the *World Allergy Organization Journal* underlined that there is evidence that FeNO failed to translate from a promising biomarker to a clinically useful tool because of a lack of understanding of confounding endogenous and exogenous factors that influence FeNO levels [31].

For epidemiological purposes, it is imperative to effectively evaluate the respiratory status of children, and therefore, it is axiomatically imperative to employ accurate assessment methods. The questionnaire serves as a vital tool, but additional measures are required to enhance its precision, as its current level of satisfaction appears unsatisfactory. In a study conducted by Kim et al., the sensitivity, specificity, and accuracy of the ISAAC questionnaire for allergic rhinitis were reported as 39.8%, 76.9%, and 63.4%, respectively [6].

## 6. Study Limitations

The present study has several limitations. Firstly, a limitation was that some children in the follow-up phase were not willing to undergo FeNO measurement. Conducting measurements during the follow-up could have enhanced the questionnaire results and confirmed the presence of elevated FeNO levels, particularly in children with a history of asthma. An additional limitation is the low response rate for the follow-up study part. In this study, the response rate was 28.6%, which is under the average value. Moreover, the corresponding study (according to methodology) performed by Czubaj-Kowal et al. had a response rate of 57.6%; however, this study had a higher sample size, and the project was realized as a part of a campaign about air pollution in Kraków [32]. Only children whose legal guardians responded via traditional post were included in the analysis. The low response rate cannot be explained by financial reasons since the letter was prepaid and payment was fully covered by the Medical University of Silesia. The possible reasons for the low response rate were the migration of the people or children who became teenagers, so they were more likely to refuse to participate in the study. The final reason for the low response rate could be the traditional letter. Legal guardians had to especially go to the post office to put letters into the letterbox. The questionnaire included only 39 questions, and there should not be a reason for such a low response rate. Unfortunately, at the same time, a low response rate could indicate selection bias.

A further limitation of the study is the possible impact of confounding factors. The anthropometric parameters were controlled, as well as the status of lung function, but environmental variables, such as exposure to tobacco smoke, severity of atopy, and physical activity, were not, which could impact the results of this study as well.

Another limitation is related to the small number of new cases of asthma; more cases of asthma could improve the investigation of the current symptoms being experienced and FeNO levels in this specific group. According to the assumptions for the sample size, it seems that the study included enough participants. With the total number of 543,231 children in the Silesia region in 2014 and the estimated prevalence of the disease at 8%, the required number of participants should be 114. However, only five new cases of asthma (without coexisting rhinitis) decrease the confidence in the results. According to the sample size estimation, there should be 10 new cases of asthma. A further problem is related to the nature of the study. The cross-sectional study on the population level should be marked with limited reliability since all symptoms are declared by legal guardians. However, the study possesses a potential strength as it is, to the author’s knowledge, the first to examine the correlation between FeNO levels and asthma-like symptoms during an 8-year follow-up. While most studies focus on prospective methods and the assessment of FeNO’s relation to the treatment control of asthma, this study offers unique insights by investigating FeNO levels regarding the presence of asthma-like symptoms over a prolonged period.

## 7. Conclusions

It seems that FeNO measurement may be a useful approach for assessing asthma prognosis in early school-aged children with asthma-like symptoms, especially from an epidemiological perspective. However, to establish its clinical validity, further confirmation is necessary by utilizing more precise diagnostic tools and informed physician decisions. Moreover, this study proves that early-age school children with a FeNO of <35 ppb are unlikely to present in the next 8 years with asthma-like symptoms.

Furthermore, adjustments should be made to the FeNO measurement method to account for influential factors like sex, age, and other allergic pathologies aside from asthma. 

Finally, further studies with larger sample sizes and with repeated measurements (prospectively repeated each year) of FeNO are needed to confirm its utility in community settings.

## Figures and Tables

**Table 1:** The mean FeNO value in relation to the status of the diagnosis at baseline and after 8 years from FeNO measurement (n = 128).

		1	2	3	4	*p* Value
Disease/Symptoms		Baseline:(−) Follow-Up:(−)	Baseline:(−) Follow-Up:(+)	Baseline:(+) Follow-Up:(−)	Baseline:(+) Follow-Up:(+)	
Asthma	FeNO [ppb]	12.8 ± 6.6	53.4 ± 75.9	21.0	37 ± 23.4	1 vs. 2 = 0.2 1 vs. 3 = n/a 1 vs. 4 = 0.09
	n	119	5	1	3	
Attacks of dyspnoea	FeNO [ppb]	14.5 ± 17.2	11 ± 1.5	25.8 ± 20.9	29.0 ± 32.5	1 vs. 2 = 0.8 1 vs. 3 = 0.3 1 vs. 4 = 0.9
	N	119	2	5	2	
Wheeze	FeNO [ppb]	12.6 ± 6.6	12.0	21.0 ± 14.5	66.7 ± 81.3	1 vs. 2 = n/a 1 vs. 3 = 0.02 1 vs. 4 = 0.09
	n	111	1	12	4	
Allergic rhinitis	FeNO [ppb]	13.3 ± 6.5	16.3 ± 12.4	12.4 ± 9.3	41.4 ± 65.3	1 vs. 2 = 0.6 1 vs. 3 = 0.1 1 vs. 4 = 0.4
	n	81	18	21	7	
Atopic dermatitis	FeNO [ppb]	13.1 ± 7.1	13.0 ± 1.7	20.0 ± 15.8	36.7 ± 66.2	1 vs. 2 = 0.5 1 vs. 3 = 0.2 1 vs. 4 = 0.7
	n	105	3	13	7	

Legend: “−“—negative status of the disease; “+”—positive status of the disease; n/a—not available.

**Table 2:** Diagnostic accuracy of FeNO at follow-up.

New Case of Outcome	FeNO [ppb]	SEN	SPEC	PPV	NPV	DOR	AUC
Asthma n = 5	>20	40.0%	81.9%	13.3%	95.1%	3.01	0.610
	>25	40.0%	94.4%	33.3%	95.7%	11.2	0.672
	>35	40.0%	98.6%	66.6%	95.9%	46.9	0.693
Allergic rhinitis n = 18	>20	16.6%	81.9%	18.7%	79.7%	3.3	0.507
	>25	16.6%	94.4%	42.8%	81.9%	3.3	0.556
	>35	11.1%	98.6%	66.7%	81.6%	8.7	0.549
Atopic dermatitis n = 3	>20	0	81.9%	0	95.1%	n/a	0.590
	>25	0	94.4%	0	95.7%	n/a	0.528
	>35	0	98.6%	0	95.9%	n/a	0.507

Legend: AUC—area under curve; DOR—diagnostic odds ratio; NPV—negative predictive value; PPV—positive predictive value; SPEC—specificity; SEN—sensitivity.

## Data Availability

The data of this study are available upon reasonable request sent to the author (Kamil Barański) of the manuscript.

## References

[1] Liebhart J., Malolepszy J., Wojtyniak B., Pisiewicz K., Plusa T., Gladysz U. (2007). Polish Multicentre Study of Epidemiology of Allergic Diseases. Prevalence and risk factors for asthma in Poland: Results from the PMSEAD study. J. Investig. Allergol. Clin. Immunol..

[2] Mazur M., Czarnobilska M., Dyga W., Czarnobilska E. (2022). Trends in the Epidemiology of Allergic Diseases of the Airways in Children Growing Up in an Urban Agglomeration. J. Clin. Med..

[3] Brozek G., Lawson J., Szumilas D., Zejda J. (2015). Increasing prevalence of asthma, respiratory symptoms, and allergic diseases: Four repeated surveys from 1993–2014. Respir. Med..

[4] Wypych-Ślusarska A., Niewiadomska E., Głogowska-Ligus J. (2022). Asthma, bronchitis respiratory symptoms, allergies and home environment: How are they related?. Adv. Dermatol. Allergol..

[5] Lai C.K.W., Beasley R., Crane J., Foliaki S., Shah J., Weiland S., ISAAC Phase Three Study Group (2009). Global variation in the prevalence and severity of asthma symptoms: Phase Three of the International Study of Asthma and Allergies in Childhood (ISAAC). Thorax.

[6] Kim D.H., Lim D.H., Samra M., Kim E.H., Kim J.H. (2018). How Accurate Are the ISAAC Questions for Diagnosis of Allergic Rhinitis in Korean Children?. Int. J. Environ. Res. Public Health.

[7] Escamilla-Gil J.M., Fernandez-Nieto M., Acevedo N. (2022). Understanding the Cellular Sources of the Fractional Exhaled Nitric Oxide (FeNO) and Its Role as a Biomarker of Type 2 Inflammation in Asthma. Biomed. Res. Int..

[8] Taylor D.R., Pijnenburg M.W., Smith A.D., De Jongste J.C. (2006). Exhaled nitric oxide measurements clinical application and in-terpretation. Thorax.

[9] Jacinto T., Malinovschi A., Janson C., Fonseca J., Alving K. (2015). Evolution of exhaled nitric oxide levels throughout development and aging of healthy humans. J. Breath Res..

[10] Pijnenburg M.W. (2019). The Role of FeNO in Predicting Asthma. Front Pediatr..

[11] Asher M.I., Keil U., Anderson H.R., Beasley R., Crane J., Martinez F., Mitchell E.A., Pearce N., Sibbald B., Stewart A.W. (1995). International study of asthma and allergies in childhood (ISAAC): Rationale and methods. Eur. Respir. J..

[12] Baraldi E., de Jongste J.C., Gaston B., Alving K., Barnes P.J., Bisgaard H., Bush A., Gaultier C., Grasemann H., Hunt J.F. (2002). Measurement of exhaled nitric oxide in children, 2001. Eur. Respir. J..

[13] Glas A.S., Lijmer J.G., Prins M.H., Bonsel G.J., Bossuyt P.M.M. (2003). The diagnostic odds ratio: A single indicator of test performance. J. Clin. Epidemiol..

[14] Prasad A., Langford B., Stradling J.R., Ho L.P. (2006). Exhaled nitric oxide as a screening tool for asthma in school children. Respir. Med..

[15] Altman D.G., Bland J.M. (1994). Diagnostic tests. 1: Sensitivity and specificity. BMJ.

[16] Pruessner J.C., Kirschbaum C., Meinlschmid G., Hellhammer D.H. (2003). Two formulas for computation of the area under the curve represent measures of total hormone concentration versus time-dependent change. Psychoneuroendocrinology.

[17] Deeks J.J. (2001). Systematic reviews in health care: Systematic reviews of evaluations of diagnostic and screening tests. BMJ.

[18] Karrasch S., Linde K., Rücker G., Sommer H., Karsch-Völk M., Kleijnen J., Jörres R.A., Schneider A. (2017). Accuracy of FENO for diagnosing asthma: A systematic review. Thorax.

[19] Caudri D., Wijga A.H., Hoekstra M.O., Kerkhof M., Koppelman G.H., Brunekreef B., Smit H.A., de Jongste J.C. (2010). Prediction of asthma in symptomatic preschool children using exhaled nitric oxide, Rint and specific IgE. Thorax.

[20] Grzelawski T., Witkowski K., Makandjou-Ola E., Grzelawska A., Majak P., Jerzyńska J., Janas A., Stelmach R., Stelmach W., Stelmach I. (2014). Diagnostic value of lung function parameters and FeNO for asthma in schoolchildren in large, real-life population. Pediatr. Pulmonol..

[21] Malmberg L.P., Pelkonen A.S., Haahtela T., Turpeinen M. (2003). Exhaled nitric oxide rather than lung function distinguishes preschool children with probable asthma. Thorax.

[22] Kovesi T., Dales R. (2008). Exhaled nitric oxide and respiratory symptoms in a community sample of school aged children. Pediatr. Pulmonol..

[23] Barański K., Zejda J. (2022). Screening Accuracy of FeNO Measurement for Childhood Asthma in a Community Setting. Children.

[24] See K.C., Christiani D.C. (2015). Normal values and thresholds for the clinical interpretation of exhaled nitric oxide levels in the US general population. Chest.

[25] Garcia-Marcos P.W., Soriano-Pérez M.J., Perez-Fernández V., Valverde-Molina J. (2016). Exhaled nitric oxide in school children: Searching for the lost variability. Allergol. Immunopathol..

[26] Barański K., Zejda J.E. (2018). Between-occasion repeatability of fractional exhaled nitric oxide measurements in children. J. Bras. Pneumol..

[27] Gochicoa-Rangel L., Rojas-Cisneros F., Miguel-Reyes J.L., Guerrero-Zúñiga S., Mora-Romero U., Maldonado-Mortera A.K., Torre-Bouscoulet L. (2016). Variability of FeNO in healthy subjects at 2240 meters above sea level. J. Clin. Monit. Comput..

[28] Kim S.H., Kim T.H., Sohn J.W., Yoon H.J., Shin D.H., Park S.S. (2010). Reference values and determinants of exhaled nitric oxide in healthy Korean adults. J. Asthma.

[29] Brody D.J., Zhang X., Kit B.K., Dillon C.F. (2013). Reference values and factors associated with exhaled nitric oxide: U.S. youth and adults. Respir. Med..

[30] Xepapadaki P., Adachi Y., Pozo Beltrán C.F., El-Sayed Z.A., Gómez R.M., Hossny E., Filipovic I., Le Souef P., Morais-Almeida M., Miligkos M. (2022). Utility of biomarkers in the diagnosis and monitoring of asthmatic children. World Allergy Organ. J..

[31] Busse W.W., Wenzel S.E., Casale T.B., FitzGerald J.M., Rice M.S., Daizadeh N., Deniz Y., Patel N., Harel S., Rowe P.J. (2021). Baseline FeNO as a prognostic biomarker for subsequent severe asthma exacerbations in patients with uncontrolled, moderate-to-severe asthma receiving placebo in the LIBERTY ASTHMA QUEST study: A post-hoc analysis. Lancet Respir. Med..

[32] Czubaj-Kowal M., Nowicki G.J., Kurzawa R., Polak M., Ślusarska B. (2022). Factors Influencing the Concentration of Exhaled Nitric Oxide (FeNO) in School Children Aged 8–9-Years-Old in Krakow, with High FeNO Values ≥ 20 ppb. Medicina.

